# Influencing factors of home exercise adherence in elderly patients with stroke: A multiperspective qualitative study

**DOI:** 10.3389/fpsyt.2023.1157106

**Published:** 2023-04-05

**Authors:** Yuanxing Zhang, Xichenhui Qiu, Qiansheng Jin, Cuiling Ji, Ping Yuan, Mengjiao Cui, Juanjuan Zhang, Lu Chen

**Affiliations:** ^1^Department of Neurosurgery, Nanjing Drum Tower Hospital Clinical College of Nanjing University of Chinese Medicine, Nanjing, Jiangsu, China; ^2^Department of Neurosurgery, Nanjing Drum Tower Hospital, The Affiliated Hospital of Nanjing University Medical School, Nanjing, Jiangsu, China; ^3^Health Science Center, Shenzhen University, Shenzhen, China; ^4^Department of Dermatology, Liuyang Hospital of Traditional Chinese Medicine, The Second Affiliated Hospital of Integrated Traditional Chinese and Western Medicine of Hunan University of Chinese Medicine, Changsha, Hunan, China

**Keywords:** elderly people, stroke, exercise adherence, influencing factor, home continuity care, qualitative study

## Abstract

**Introduction:**

Evidence has shown that stroke exercise rehabilitation is the most effective way to improve disease prognosis, but home exercise adherence in elderly patients with stroke is low due to they are more likely to have movement disorders, cognitive disorders, mental disorders, etc. Currently, most studies on exercise adherence in elderly patients with stroke are quantitative, and there is a lack of qualitative studies from the perspective of patients, caregivers, and medical staff. Considering the importance of home exercise adherence in elderly patients with stroke, the present study aimed to explore the influencing factors of home exercise adherence in them and summarize the potential ways to improve it.

**Methods:**

From October to December 2022, 9 medical staff, 12 elderly patients with stroke and 7 caregivers from a level A tertiary hospital and community health service center in Nanjing, Jiangsu Province were selected by the purposive sampling and were interviewed in a face-to-face semi-structured way. The data were analyzed and summarized by the phenomenological analysis of Colaizzi’s method.

**Results:**

The influencing factors of home exercise adherence in elderly patients with stroke can be summarized into 3 themes and 8 subthemes. These were individual factors (physical impairment, exercise self-efficacy, and depression), family factors (caregiving ability and emotional support); and stroke rehabilitation environment (exercise prescription, monitoring and feedback, and organizational policy).

**Conclusion:**

Home exercise adherence in elderly patients with stroke was influenced by many factors. Medical staff should assess the patient’s physical function and depression, establish a multi-support system, formulate personalized exercise prescription, pay attention to the monitoring and feedback of home-based exercise rehabilitation, and improve the home-based rehabilitation model for stroke, so as to improve the home exercise adherence in elderly patients with stroke and promote the best rehabilitation effect.

## Introduction

Worldwide, stroke is one of the most serious diseases threatening human life and health today; it has risen to become the second leading cause of death and the third leading cause of disability (1). In recent years, the prevalence of stroke in China has increased year by year, ranking first in the world, due to changes in people’s lifestyle and the ageing of the population and other factors. It has become one of the leading causes of death in China and is most commonly observed in elderly people (2, 3). In addition, 70–80% of patients have varying degrees of limb motor and cognitive dysfunction after a stroke (4). Post-stroke hemiplegia, anxiety, and depression can trigger a chain reaction in which elderly patients with stroke are severely limited in their ability and motivation to participate in long-term exercise rehabilitation (5). As a result, disability and death caused by stroke can greatly increase the burden on families and society.

According to evidence-based medicine, stroke rehabilitation is the most effective way to reduce the rate of disability and restore various functions in patients (6). Providing exercise rehabilitation to elderly patients with stroke sequelae is essential to improve various limb functions and enhance their quality of life (7, 8). Exercise rehabilitation is the preferred rehabilitation treatment for stroke patients; however, the exercise adherence of these patients is generally low (9). Exercise adherence refers to the degree to which an individual adheres to an established exercise prescription. It reflects the patient’s level of participation in exercise rehabilitation and the level of rehabilitation, which can be simply understood as the patient’s adherence with his or her exercise prescription (10). Due to various policy, social and family factors, long-term inpatient rehabilitation is difficult for stroke patients, and home rehabilitation is often the first choice, and it has proven to be functionally and cost effective in improving patients’ limb function, activities of daily living and quality of life and reducing disease recurrence rates (11). It was found that 63 to 82% of stroke patients had good adherence during hospitalization, but only 47.41% of patients showed good adherence after discharge. The exercise adherence curve was in the shape of a horizontal “S” and changed over time: the adherence was better at the beginning of the disease, decreasing before discharge, and significantly decreasing after discharge (12, 13). As most elderly patients with stroke are not sufficiently aware of functional exercise after discharge from hospital, poor exercise adherence not only affects patients’ self-care ability, but also reduces quality of life (14). How to promote home exercise adherence in elderly patients with stroke has become a focus of research. Therefore, it is important to identify factors that influence exercise adherence in elderly patients with stroke and to design precise scientific interventions.

Current factors influencing exercise adherence in stroke patients include ability (physical and mental ability), opportunity (personal and social), and motivational (reflection and reinforcement) (15–17). Most of the qualitative studies on the factors influencing exercise adherence include only a single aspect of the patient, and there is a lack of studies on home exercise adherence in elderly patients with stroke and several studies involving patients, their caregivers, and medical staff (15–17). Therefore, in this study, we analyzed the factors influencing the exercise adherence in elderly patients with stroke through face-to-face semi-structured interviews with patients, caregivers, and medical staff to provide a reference for improving the home-based stroke rehabilitation model.

## Methods

### Design

In accordance with the purpose of the study, a descriptive phenomenological method was used in this study. Descriptive phenomenology requires observing a particular phenomenon, distilling the core elements of the phenomenon, and exploring the elements and their relationship to the surrounding scenario (18). In Husserl’s descriptive approach, researchers are asked to focus on the participants’ experience of a phenomenon and determine the nature of the phenomenon while setting aside their own beliefs, attitudes, previous experiences, and assumptions (19). The experience of home exercise rehabilitation in elderly patients with stroke is influenced by factors such as interpersonal communication and the environment, and is appropriate for this method. The Consolidated Criteria for Reporting Qualitative Research (COREQ) was used to report the findings (20).

### Participants

From October to December 2022, 9 medical staff, 12 elderly patients with stroke and 7 caregivers from a level A tertiary hospital and community health service center in Nanjing, Jiangsu Province were selected by the purposive sampling combined with a maximum variation sampling (21) and were interviewed in a face-to-face semi-structured way. The inclusion criteria for medical staff were (1) at least 5 years in hospital neurology and rehabilitation, and at least 5 years in the community; (2) intermediate and above title; (3) informed consent to participate in this study. The exclusion criteria for medical staff were (1) absent on leave; (2) refresher doctor or nurse. The inclusion criteria for patients were (1) meet the standards established by the Fourth Chinese Symposium on Cerebrovascular Disease in 1995 and be diagnosed with stroke by CT or MRI; (2) first stroke; (3) duration ≥3 months, clear consciousness to cooperate with the study and stable vital signs; (4) over 60 years old. The exclusion criteria for patients were (1) severe heart, liver, kidney, and other organ dysfunction and malignancy; (2) severe aphasia, or cognitive dysfunction; (3) participation in other research programs. The inclusion criteria for caregivers were (1) a relative of the patient and not required to provide care costs; (2) over 18 years old; (3) literate enough to understand and cooperate with the study. The exclusion criteria for caregivers were cognitive, speech, or mental problems. The sample size in this study was determined according to the principle of “information saturation,” i.e., the interview data of each of the three types of subjects reached saturation, and no new themes or subthemes emerged during the interview process. The study was approved by Medical Ethics Committee of Nanjing Drum Tower Hospital, the Affiliated Hospital of Nanjing University Medical School (No.2021-381-01). All study subjects gave informed consent and signed an informed consent form. The general information of the study subjects are shown in Tables 1–3.

**Table 1:** Medical staffs’ characteristics (*n* = 9).

Number	Gender	Age (years)	Education	Professional title	Position	Working years (years)
A	Female	40	Bachelor	Intermediate	Community doctor	16
B	Female	39	Bachelor	Intermediate	Community nurse manager	16
C	Male	51	Doctor	Senior	Doctor	23
D	Female	38	Master	Intermediate	Rehabilitation therapist	13
E	Male	35	Master	Intermediate	Rehabilitation therapist	11
F	Female	49	Bachelor	Senior	Stroke follow-up center outpatient nurse	26
G	Female	40	Master	Intermediate	Nurse manager	15
H	Female	35	Master	Intermediate	Nurse manager	9
I	Male	35	Bachelor	Intermediate	Nurse	13

**Table 2:** Patients’ characteristics (*n* = 12).

Number	Gender	Age (years)	Education	Occupation	Marital status	Monthly household income (thousand Yuan)	Medical insurance	Type of stroke	Course of disease (month)
P1	Male	62	Elementary school	Freelancer	Married	≥10	Have	Ischemic stroke	6
P2	Male	65	Elementary school	Freelancer	Divorced	≥10	Have	Hemorrhagic stroke	7
P3	Female	77	Middle school	Waiter	Married	5–10	Have	Ischemic stroke	8
P4	Female	68	High school	Veteran	Married	5–10	No	Ischemic stroke	7
P5	Female	67	Elementary school	Farmer	Unmarried	5–10	No	Hemorrhagic stroke	5
P6	Male	80	High school	Teacher	Married	5–10	Have	Ischemic stroke	8
P7	Female	71	Middle school	Nurse	Divorced	≥10	Have	Ischemic stroke	4
P8	Female	70	Elementary school	Farmer	Married	≥10	Have	Hemorrhagic stroke	10
P9	Male	73	Middle school	Freelancer	Married	≥10	Have	Ischemic stroke	4
P10	Male	65	College	Teacher	Married	≥10	Have	Ischemic stroke	5
P11	Female	68	Elementary school	Construction worker	Widowed	5–10	No	Ischemic stroke	12
P12	Female	72	High school	Civil servant	Divorced	5–10	Have	Hemorrhagic stroke	5

P: patient.

**Table 3:** Caregivers’ characteristics (*n* = 7).

Number	Gender	Age (years)	Education	Monthly household income (thousand Yuan)	Relationship with patients	Duration of caregiving (month)
C1	Male	67	Elementary school	5–10	Couples	7
C2	Female	65	Middle school	5–10	Couples	6
C3	Female	34	High school	5–10	Mother-daughter	5
C4	Male	70	Middle school	≥10	Couples	7
C5	Male	35	College	≥10	Father-son	5
C6	Female	67	High school	≥10	Couples	5
C7	Female	78	Elementary school	5–10	Couples	8

C: caregiver.

### Data collection

The literature was searched according to the purpose of the study, and a preliminary interview outline was developed by subject matter experts with experience in qualitative research. Two cases each of medical staff, patients, and caregivers who met the inclusion and exclusion criteria were selected for pre-interviews, and hard-to-understand content of the interview outline was revised to form a formal interview outline. To identify domains, we developed three to five open-ended questions for each of the three groups (Box 1). During the interviews with the medical staff, the main question was related to their perspectives concerning patients’ home exercise rehabilitation and their experiences of teamwork. While interviewing the patients, their experiences of home exercise rehabilitation and the facilitators and barriers were explored. Finally, the caregivers were asked about their expectations and needs for cooperation in care as well as the existing problems in this field. Data were collected by two researchers using semi-structured interviews and observational methods, who received professional training in qualitative research methods prior to the interviews to ensure the rigor of the data collection process. Patients and their caregivers were selected to be interviewed after the outpatient visit in a location chosen to be an undisturbed demonstration room, where patients and caregivers were interviewed separately and individually. Interviews with medical staff were conducted in their departments. The researcher introduced himself before the interview, explained the purpose and methods of this study to the subjects, and signed the informed consent form after obtaining their consent; the interview time should be determined according to the situation, and the interview process was recorded. During the interview, the researcher should listen carefully and use communication skills to relieve the patient’s tension, ask in-depth questions and clarifications based on the scenario, and avoid subjective guidance by the researcher. The researcher should pay attention to the non-verbal characteristics such as the patient’s expression, movement and speech rate during the interview and take notes of the interview. The interviews were stopped when no new content emerged from the interview results, i.e., the data were saturated, and the subjects were referred to by number to protect their privacy. In this study, the duration of interviews with medical staff, patients and caregivers was 15–19 min, 23–48 min, and 18–27 min, respectively.

**Box 1**

Open-ended questions tailored to participants’ groups (medical staff, caregivers, and patients).

Medical staff.

1. “What is your hospital doing to improve home exercise adherence in older patients with stroke?”
2. “What are your recommendations for managing patients’ home exercise rehabilitation?”
3. “What do you think are the facilitators and barriers for patients to engage in home exercise rehabilitation?”

Patients.

1. “Do you follow your healthcare provider’s instructions to exercise?”
2. “What motivates you to exercise/why don’t you exercise at home?”
3. “Did you encounter any difficulties during your home exercise rehabilitation and how did you solve them?”
4. “What help did you receive during your home exercise rehabilitation?”
5. “How did you feel during your home exercise rehabilitation?”

Caregivers.

1. “Do you follow medical advice to monitor and supervise your patients’ home rehabilitation behaviors?”
2. “What do you think are the difficulties you encounter in managing patients’ home rehabilitation?”
3. “Do you seek help actively when you are in trouble? What are the sources of help you get?”

The continuation of the interview will be based on participatory answers and exploratory questions.

### Data analysis

Within 24 h of the interviews, the audio recordings were transcribed by two researchers independently, and the data were analyzed simultaneously by two researchers using Colaizzi’s seven-step phenomenological analysis to clarify thematic relationships at different levels (22). This method provides a rigorous analysis, with each step staying close to the data: (1) read all collected transcriptions carefully and repeatedly; (2) identify relevant, important, or meaningful statements and prepare memos; (3) code recurring ideas; (4) cluster initial themes by looking for common codes; (5) describe themes in detail and select appropriate statements for quotations; (6) produce and refine themes and subthemes; and (7) ask interviewed participants for confirmation (Ten participants were randomly chosen for verification in this study). The transcripts were managed and analyzed with NVivo 12 software, a Computer-Assisted Qualitative Data Analysis Software (CAQDAS) (23). A third researcher was consulted in case of disagreement.

### Quality control method

Before data collection, trust relationship was established with medical staff in the department to obtain support and cooperation. In the process of encoding data, the interviewers always reflect on themselves, try to straighten out their own position, explore and grasp the experience, process or culture studied from the perspective of the interviewees, and do not substitute their own unique background, values, social and professional identity into the research. After each interview, the researcher wrote a reflective journal to ensure the quality and objectivity of the interview.

## Results

Three main themes and subthemes were extracted from the analysis of data that included individual factors, family factors, and stroke rehabilitation environment (Table 4).

**Table 4:** Themes and subthemes.

Themes	Subthemes
Individual factors	Physical impairment
	Exercise self-efficacy
	Depression
Family factors	Caregiving ability
	Emotional support
Stroke rehabilitation environment	Exercise prescription
	Monitoring and feedback
	Organizational policy

### Theme 1: Individual factors

Individual factors were found to be one of the most important factors influencing home exercise adherence in elderly patients with stroke. This theme consisted of three subthemes, namely physical impairment, exercise self-efficacy, and depression.

#### Physical impairment

Physical impairments include sensory and motor impairments that may prevent patients from performing home exercise rehabilitation, including muscle weakness, spasticity, and pain.

*“I want to exercise too, but I have no strength in my legs and have to hold them up to stand up, let alone walk (sigh).” (P6 and P10)*

*“In my home rehabilitation, I sometimes have unconscious spasms in my arms and legs, and I have to rest every time I have a spasm, so it's hard to follow the exercise plan.” (P12)*

*“The most he reflects at home exercise is pain, either screaming headache or body pain, every time he is in pain, I can not bear to let him continue to exercise, and do not know when to recover.” (C1 and C5)*

#### Exercise self-efficacy

Good exercise self-efficacy can mobilize patients’ initiative, increase motivation and self-confidence in exercise rehabilitation, and thus enhance home exercise adherence.

*“I usually get up at 5 a.m., cook the porridge, and then go to the community square to do Tai Chi for half an hour, no time wasted; and no time to squeeze in.” (P5)*

*“When the disease first appeared, I felt very helpless, but then I thought I couldn't give up, there was still a long way to go, and I had to believe that if I kept exercising, I would get back to my previous state of life as soon as possible.” (P7 and P8)*

However, patients with low exercise self-efficacy lack the confidence to overcome difficulties when they encounter them, resulting in poor exercise rehabilitation behaviors.

*“I know that more exercise is good for functional recovery, but I tend to back off when I have a little difficulty exercising at home and can't continue.” (P1)*

*“She rarely exercised before she got sick, and now that she's had a stroke, she doesn't want to exercise, is more anxious, and just wants to lie down at home every day.” (C4)*

#### Depression

Elderly patients with stroke experience depressive symptoms such as low mood due to the illness, which significantly reduces home exercise adherence.

*“I've been unhappy lately, not interested in anything, and I don't want to exercise; I'm sorry to my family.” (P2, P4 and P11)*

*“He was particularly pessimistic and felt there was no hope for him to live; lately he had trouble eating and sleeping, often waking up in the middle of the night.” (C7)*

*“Some of our patients have depression, and the elderly are very vulnerable after a stroke, not to mention exercising at home.” (A, B and E)*

### Theme 2: Family factors

Family factors play an important role in the home exercise adherence in elderly patients with stroke, including the capacity of family members to provide care and emotional support.

#### Caregiving ability

The caregiver’s caregiving ability affects the patient’s disease prognosis, and the higher the caregiver’s caregiving ability, the higher the home exercise adherence in elderly patients with stroke.

*“Since my onset, my wife has taken care of my daily routine and also accompanies me for exercise and review, so I am on a daily exercise schedule and currently have no after-effects.” (P9)*

*“Since she came home from the hospital, I will remind her every day on time to take her medication, exercise ah, there is nothing you can do with this disease, but she will do what the doctor said so far.” (C3)*

#### Emotional support

Most patients reported that the support of their families was a strong motivation for them to persevere with their home rehabilitation.

*“My family and children are very supportive of my training. My family stayed with me when I started exercising because they were afraid I would fall; my family also took me to the hospital for regular review, they worked so hard and I just wanted to recover as soon as possible.” (P2)*

*“Since I've been sick, my sister will call the video to take care of me, I'm very touched by it, all I can do is to actively exercise, keep a positive attitude and return to normal life as soon as possible.” (P7)*

*“Strengthening family support is especially important in the elderly population, where sudden stroke can have a major impact on daily life, and it is necessary to assess family members' willingness to care and provide appropriate interventions.” (G and I)*

### Theme 3: Stroke rehabilitation environment

The stroke rehabilitation environment is a health care and policy factor that influences home exercise adherence in elderly patients with stroke, including exercise prescription, monitoring feedback, and organizational policy.

#### Exercise prescription

Exercise prescription that is straightforward, safe, feasible, and consider patient preferences can improve patient’s home exercise adherence.

*“Before I was discharged from the hospital, there was a special rehabilitation therapist who helped me develop an exercise rehabilitation program and sent me some rehabilitation videos that I was willing to follow at home; at the same time, when I was reviewed, the medical staff would adjust the exercise movements and time according to my specific situation.” (P9)*

*“My exercise program was developed by my doctor according to my physical condition, it is scientific and safe, I am willing to exercise.” (P6)*

The absence or abstraction of an exercise prescription makes it difficult to practice and reduces patient’s home exercise adherence.

*“When I was discharged from the hospital, I asked my doctor how I should exercise at home, but he just told me to walk every day, but I still didn't know exactly how long I needed to walk to see effect.” (P1)*

*“When my family was hospitalized, there was no special doctor to develop an exercise program, now we search the Internet for exercise videos, and I do not know if it is right.” (C5 and C6)*

#### Monitoring and feedback

It is beneficial to improve the quality of home rehabilitation by increasing the monitoring and feedback of stroke patients during exercise. Sources of monitoring and feedback may include family members, community members, and medical staff.

*“When I was in the hospital, the nurses urged me to move more every day, and my family actively supervised me; after I returned home, sometimes when I was lazy and didn't want to move, my family would chase me away, just hoping I would recover faster.” (P2 and P3)*

*“Because my condition was serious, I was seen by a community physician at home when I first came home, and my recovery was monitored regularly thereafter, and I was able to ask questions and solve problems in a timely manner.” (P6)*

*“It is necessary to strengthen patient exercise monitoring in many aspects. I have encountered many problems in my work, and many patients are able to do better exercise while in the hospital, but most of them have difficulty maintaining better exercise habits after they go home.” (D, F)*

#### Organizational policy

The policy system is the prerequisite for patient safety and the basis for quality assurance, but there is no comprehensive health insurance policy, stroke exercise rehabilitation construction and cooperation mechanism among various disciplines and medical institutions, and standardized organizational policies are still needed.

*“Most of the money is spent on surgery and medication, and there is no medical insurance for stroke rehabilitation, so there is no money for rehabilitation.” (C2)*

*“To do a good job of home exercise rehabilitation for stroke patients, it cannot be done by our medical staff alone, but requires government leadership, as well as institutional and legal safeguards.” (H)*

*“At present, the hierarchy of responsibilities among the three levels of medical institutions is unclear, and there is no clear positioning of what each level of hospital should be responsible for; at the same time, there is a lack of multidisciplinary collaborative management in home stroke rehabilitation.” (C)*

## Discussion

The present study aimed to identify the influencing factors of home exercise adherence in elderly patients with stroke based on the experiences of patients, caregivers, and medical staff. The factors that were derived to influence home exercise adherence included individual factors, family factors, and stroke rehabilitation environment. More often than not, several of the three themes were related and interwoven together, as has also been reported in previous studies.

In this study, physical impairment and psychological status were the most profound experiences of patients in home exercise rehabilitation. The results showed that muscle weakness, spasticity, and pain decreased patients’ home exercise adherence, which is consistent with the findings of Damush et al. (24). Stroke patients have declining muscle function, which affects their mobility, and severe physical impairment makes it difficult for them to perform regular exercise (25). It is recommended that medical staff develop intervention strategies for stroke patients with declining muscle function and improve cognitive management of home spasticity and pain in patients for early intervention. In addition, this study found that improved exercise self-efficacy has a facilitating effect on home exercise rehabilitation in elderly patients, which is consistent with the findings of Caetano et al. (26). The patient’s cognitive appraisal of the disease may be an important support mechanism for patient adherence to long-term exercise rehabilitation, and the virtuous circle it creates may increase the patient’s exercise self-efficacy (27). The results also suggest that low exercise self-efficacy reduces patients’ motivation to exercise. This suggests that medical staff should guide patients to stimulate positive emotions and use behavior change strategies to enhance their exercise self-efficacy, thereby promoting their home exercise adherence.

The results of this study show that elderly patients with stroke are prone to depressive symptoms that reduce their intrinsic motivation to exercise, which is consistent with the findings of Morris et al. (28). The body’s nervous, endocrine and immune systems can become dysregulated or overactivated through maladaptation in response to stress, leading to adverse health behaviors (29). The elderly are in a gradual aging process, with physical function gradually declining, they are more likely to become depressed when suffering from illness (30). Therefore, medical staff should enhance the early detection of depressive symptoms in elderly patients with stroke for early psychological and pharmacological treatment to enhance motor motivation.

The caregiver’s ability to care for the patient influenced the patient’s disease prognosis, and that support and supervision from family members and medical staff can influence home exercise in elderly patients with stroke positively. Harrison et al. (16) found that multiple support was a key factor contributing to patients’ home exercise adherence and that patients who received exercise supervision had higher exercise adherence. Family members’ companionship and supervision increase patients’ confidence in exercise rehabilitation, while medical staff supervision ensures scientific and safe exercise procedures. This suggests that medical staff need to provide adequate education and support to not only patients, but also to their caregivers on topics such as autonomous supportive environment and problem solving to comprehensively enhance caregivers’ willingness and ability to play an active role in patients’ home rehabilitation. With the advent of artificial intelligence, wearable sensor devices can be used to monitor patients’ home exercise feedback, including weekly exercise frequency, intensity, time, and number of repetitions, and provide risk warnings based on patients’ exercise parameters to ensure home exercise safety (31).

This study found that most elderly patients with stroke were aware of the specific form, frequency, duration, and intensity of home exercise rehabilitation, but some were unable to accurately grasp the exercise prescription, which is consistent with the study by Jansson et al. (15). The significant positive predictive effect of a well-established exercise prescription on patient exercise adherence has been demonstrated in a previous study (32). Exercise prescription is influenced by subjective norms, behavioral attitudes, and perceived behavioral control, and individualized exercise prescription can improve patients’ exercise participation mastery and adherence (33). Therefore, medical staff should consider the patient’s physical function, exercise preference, and rehabilitation readiness, and help the patient formulate reasonable goals and personalized prescriptions to improve the science and feasibility of exercise prescription.

The current stroke exercise rehabilitation system is not well developed, and there is a shortage of community professionals and inadequate cooperation among medical institutions. The cost of stroke rehabilitation is an important factor affecting the home exercise adherence in elderly patients, and the elderly population has difficulty in adhering to review and community rehabilitation because they do not have stable financial resources (34). It is recommended that the government should develop stroke rehabilitation policies with service innovations that take into account the characteristics of the elderly population, and provide rehabilitation services that are different from those of younger patients. Different levels of medical institutions also need to clarify their responsibilities and cooperate with each other to build a smooth and efficient management system and operation mechanism. How to build an economical, safe, and scalable home-based exercise rehabilitation model for the elderly is a direction worthy of in-depth study.

### Limitations and strong points

This research study was one of the first qualitative studies that assessed the viewpoints of medical staff, patients, and caregivers in terms of facilitators and barriers of home exercise adherence in elderly patients with stroke. This study focuses specifically on the key stakeholders involved in patient rehabilitation at home, which is useful for managers and policymakers to use in care programs. However, this study had some limitations. The limitation of this study is that the respondents were from only one region. Although the data was saturated, some surprising and meaningful statements or themes might emerge if more participants from different regions were interviewed. In the future, a mixed-methods design in qualitative research is expected to help achieve impartiality. The simultaneous use of quantitative and qualitative techniques can bring balance to qualitative research and thus achieve equity. Future studies can expand the scope of the study and include the type of stroke into the quantitative study to study the impact of stroke type and other factors on home exercise adherence in elderly patients with stroke, so as to further refine the findings.

## Conclusion

This qualitative study used a descriptive phenomenological method to analyze the factors influencing home exercise adherence in elderly patients with stroke, resulting in three themes and eight subthemes of individual factors, family factors and stroke rehabilitation environment. Medical staff should pay attention to patients’ physical impairment and depression, and establish a multiple emotional support system, pay attention to home exercise monitoring feedback, and develop precise exercise prescriptions. The government should pay more attention to elderly patients with stroke and improve the home exercise rehabilitation system for the elderly. This study may provide evidence for the development of intervention strategies to improve home exercise adherence in elderly patients with stroke, thereby promoting optimal recovery.

## Data availability statement

The raw data supporting the conclusions of this article will be made available by the authors, without undue reservation.

## Ethics statement

The studies involving human participants were reviewed and approved by Medical Ethics Committee of Nanjing Drum Tower Hospital, the Affiliated Hospital of Nanjing University Medical School (No. 2021-381-01). The patients/participants provided their written informed consent to participate in this study.

## Author contributions

YZ, XQ, QJ, JZ, and LC contributed to the study conception and design. Material preparation, data collection and analysis were performed by YZ, CJ, PY, and MC under the supervision of JZ and LC. The first draft of the manuscript was written by YZ and XQ. All authors contributed to the article and approved the submitted version.

## Funding

This work was supported by the Postgraduate Research & Practice Innovation Program of Jiangsu Province (grant number SJCX22_0815) and fundings for Clinical Trials from the Affiliated Drum Tower Hospital, Medical School of Nanjing University (grant number 2021–LCYJ–MS–05).

## Conflict of interest

The authors declare that the research was conducted in the absence of any commercial or financial relationships that could be construed as a potential conflict of interest.

## Publisher’s note

All claims expressed in this article are solely those of the authors and do not necessarily represent those of their affiliated organizations, or those of the publisher, the editors and the reviewers. Any product that may be evaluated in this article, or claim that may be made by its manufacturer, is not guaranteed or endorsed by the publisher.
